# A Case of Ectopic Extramammary Paget’s Disease on the Forehead

**DOI:** 10.7759/cureus.112609

**Published:** 2026-07-13

**Authors:** Nathan Jetter, Adriana Pierangeli, Karla Dunning, Vassilios Dimitropoulos

**Affiliations:** 1 Dermatology, Clear Choice Dermatology, Federal Way, USA; 2 Dermatology, St Joseph Dermatology and Vein Clinic, St Joseph, USA; 3 Pathology, Pathology Services of Kalamazoo, Kalamazoo, USA

**Keywords:** cutaneous oncology, dermatopathology, empd, extramammary paget’s disease, immunohistochemistry staining, mohs surgery, primary extramammary paget’s disease

## Abstract

We report a case of ectopic primary extramammary Paget’s disease (EMPD) on the forehead with positive immunohistochemical staining for cytokeratin-7 (CK7). Primary EMPD is an uncommon intraepidermal adenocarcinoma arising in the skin, primarily in apocrine gland-rich areas, most commonly in the genital and perianal regions. To our knowledge, this is the first case of EMPD reported on the forehead. There have only been four prior cases of ectopic primary EMPD reported on the face, and we provide an updated table of the prior and related cases.

## Introduction

Primary extramammary Paget’s disease (EMPD) is an uncommon intraepidermal adenocarcinoma arising in the skin. The majority of primary EMPD lesions are located in apocrine gland-rich areas, commonly in the genital and perianal regions. EMPD is classified as secondary when it results from extension of an underlying adenocarcinoma into the skin. EMPD’s prognosis is generally favorable because the tumor stays for a prolonged duration in the radial growth phase, and a majority of EMPD lesions are treated with margin-controlled surgery while the tumor is still in situ. Though uncommon, EMPD does have metastatic potential, particularly once it invades the dermis [1-3].

Primary EMPD that arises in non-apocrine gland-bearing regions is rare and has been described as “ectopic” EMPD [1,2]. We report a case of ectopic EMPD of the forehead. To our knowledge, this is the first case of EMPD reported on the forehead. There have only been four prior cases of ectopic primary EMPD reported anywhere on the face, and three additional cases reported as extending to the face from the oral mucosa or the glands of Moll of the eyelid [2,4-7].

## Case presentation

**Figure 1:**
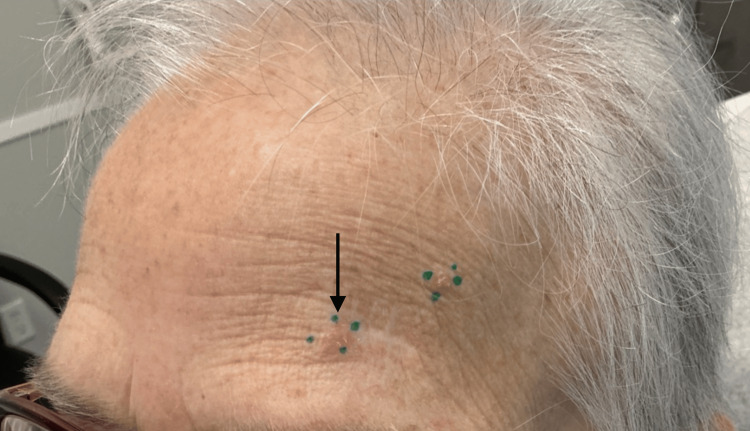
The left medial inferior forehead lesion indicated by the arrow is the EMPD. The left lateral superior forehead lesion was biopsied as well and was reported as BLK. EMPD: extramammary Paget's disease; BLK: benign lichenoid keratosis.

A 71-year-old female presented with an erythematous, scaly, poorly demarcated plaque on the left forehead. The slightly raised lesion was located approximately 1 cm inferior to a scar from a Mohs micrographic surgery (MMS) procedure for cutaneous squamous cell carcinoma (cSCC) performed two years earlier. The lesion measured 1.5 x 0.9 cm. The patient noticed the lesion one year prior to presentation, and the lesion was initially treated with liquid nitrogen cryotherapy for presumed actinic keratosis. When the lesion failed to resolve following liquid nitrogen cryotherapy, it was subsequently biopsied due to concern of recurrent cSCC (Figure 1). EMPD was not considered in the differential diagnosis at the time of biopsy.

**Figure 2:**
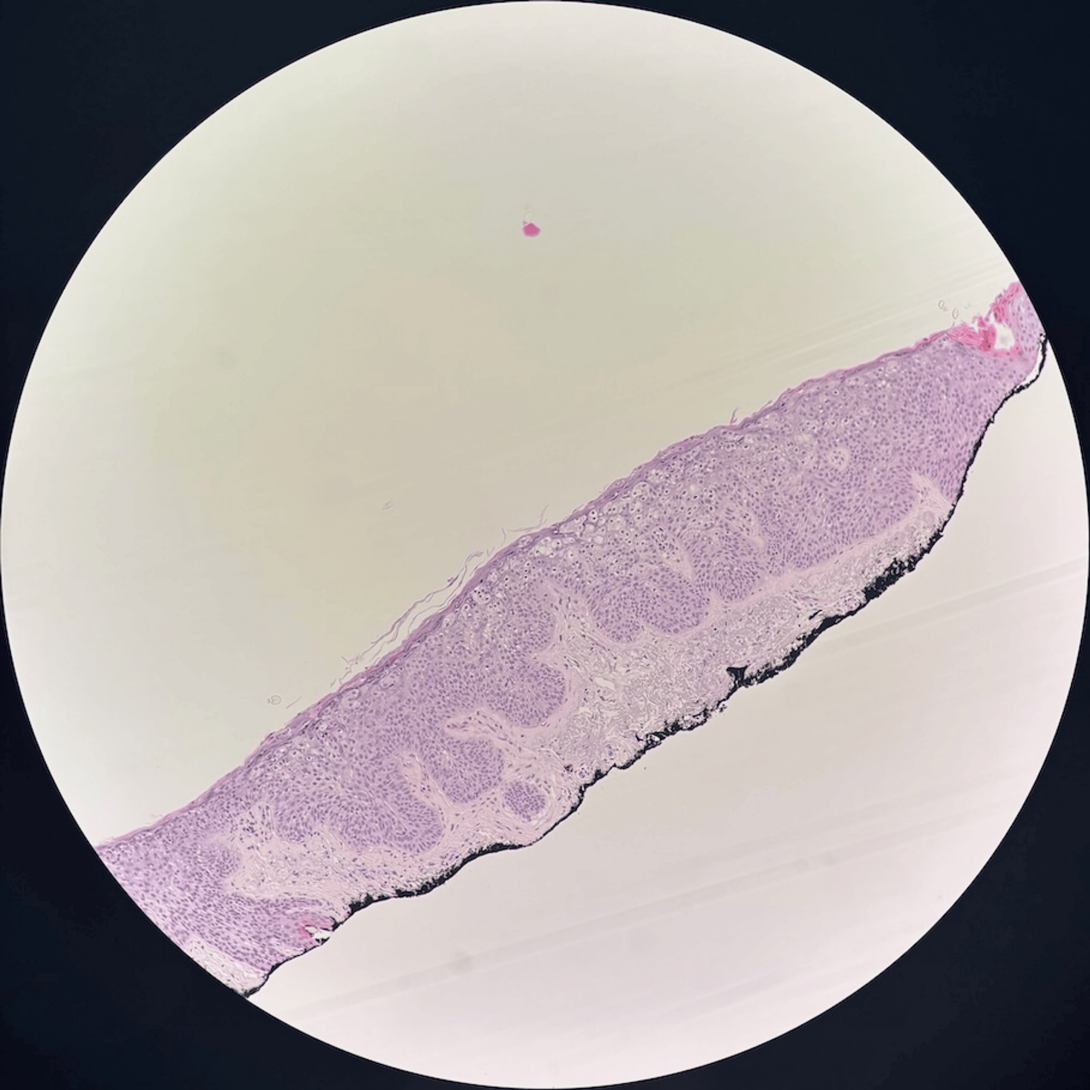
Hematoxylin and eosin (H&E) histopathology.

**Figure 3:**
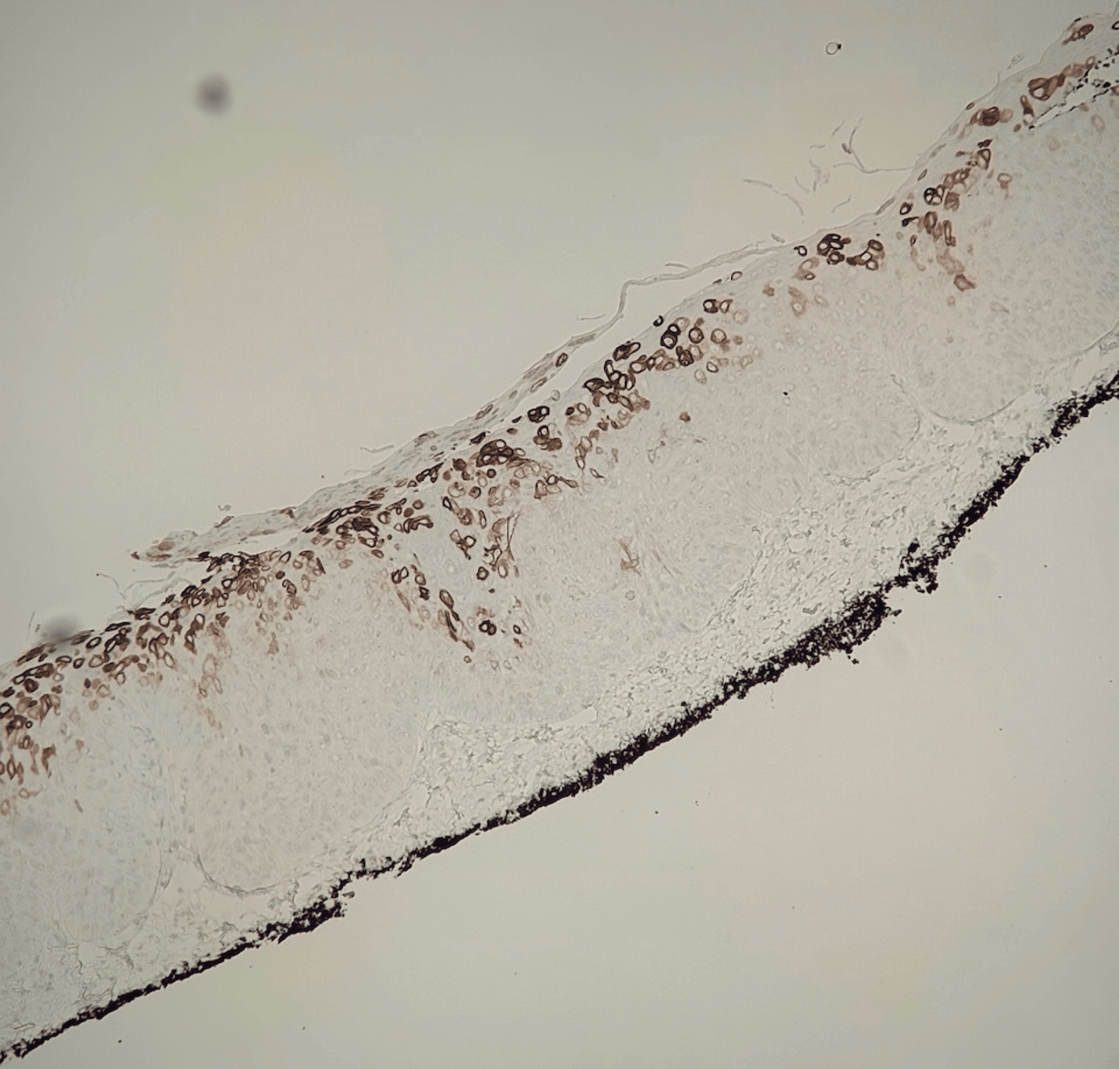
Cytokeratin-7 (CK7) immunohistochemistry.

Pathology demonstrated a pagetoid spread of atypical pale epithelioid cells in the epidermis with hypergranulosis, parakeratosis, and hyperkeratosis (Figure 2). Immunohistochemical (IHC) staining was performed to further elucidate the diagnosis. Cytokeratin-7 (CK7) marked the cells of interest (Figure 3). Tumor protein p63 appropriately stained background keratinocytes. Lesional cells were negative for SRY-box transcription factor 10 (SOX-10) and Melan-A, both of which were appropriately positive in rare basally oriented background melanocytes. The specimen was sent to Mayo Clinic for expert consultation, where additional IHC staining demonstrated positive epithelial membrane antigen (EMA) and negative gross cystic disease fluid protein-15 (GCDFP-15) of the Paget cells. Taken together, the histopathologic and immunohistochemical findings were consistent with primary EMPD occurring on the forehead.

The patient underwent treatment of their EMPD by MMS, and the lesion was cleared in one layer. A post-MMS margin was sent for permanent section pathology confirmation and was found to be clear of residual neoplasm. Lymph nodes were normal on exam, and review of systems did not reveal symptoms concerning internal malignancy. The patient was referred to her primary care doctor and to oncology for full age- and sex-appropriate cancer screening.

## Discussion

In 1874, Paget's disease was first described as a skin cancer of the nipple. In 1889, EMPD was identified in the scrotum and genital area [1]. Primary EMPD occurs in areas with a high concentration of apocrine sweat glands, mostly in the axillary and genital regions [4]. Ectopic cases outside these areas are rare, especially on the face, with only four prior cases occurring on the face reported in the English literature (Table 1). Three of the prior facial primary EMPD cases, located on the cheek, nose, and lateral canthus, were treated with Mohs surgery. The remaining case, also located on the cheek, was treated with wide local excision (WLE) [4-7].

**Table 1:** Previously reported cases of facial EMPD. EMPD: extramammary Paget’s disease.

	Article & year	Location
Ectopic primary EMPD of the face	Hughes et al. (2019) [4]	Nose
	Ximena et al. (2012) [5]	Cheek
	Cohen et al. (2004) [6]	Lateral canthus
	Chilukuri et al. (2002) [7]	Cheek
EMPD extending to the face from oral mucosa or Moll’s glands	Wang et al. (2017) [2]	Oral mucosa, perioral skin
	Knauer et al. (1963) [8]	Moll’s gland, eyelid
	Whorton et al. (1955) [9]	Moll’s gland, eyelid

We also note that three cases of EMPD have been reported extending to facial skin from oral mucosa (one case) and Moll’s glands (two cases) [2,8,9]. Cases of EMPD arising from Moll’s glands are generally considered primary non-ectopic EMPD because Moll’s glands are a modified type of apocrine gland [2,10]. There are no true or modified apocrine glands in normal human oral mucosa; human salivary glands are merocrine. That being said, the term “ectopic” has generally not been used in the literature describing EMPD arising from oral mucosa [2,11].

EMPD has a peak incidence between 45 and 75 years of age. It is most common in Caucasian patients, with a male-to-female ratio of 1:2-7 [1]. Ectopic EMPD lesions in non-apocrine areas, such as in our patient, are often misdiagnosed for extended periods because of their potential resemblance to other conditions. Reports of erroneous inflammatory and infectious clinical diagnoses include eczema, psoriasis, lichen simplex chronicus, lichen planus, pemphigus vegetans, and candidiasis. EMPD has been mistakenly diagnosed as neoplastic entities, including cSCC, as in our case, basal cell carcinoma, and mycosis fungoides [1,12]. Misdiagnoses at initial presentation commonly lead to treatment delays. EMPD lesions are biopsied after an average of one to three years [5].

Positive IHC staining with CK7 is a classic marker for EMPD. Additional staining with SOX10, Melan-A, human melanoma black 45 (HMB-45), and S-100 can help differentiate EMPD from melanoma [6]. EMA and GCDFP-15 are markers of apocrine differentiation [1,2]. There is evidence that suggests GCDFP-15 positivity is associated with primary EMPD and may be positive in up to 90% of primary EMPD. Our case is in the minority of ectopic EMPD cases, which are primary but lack GCDFP-15 positivity. GCDFP-15 positivity may also have high specificity for the absence of a related internal malignancy, although this does not necessarily translate into sensitivity for internal malignancy. GCDFP-15 may have value when used in a panel of IHC stains for establishing the source of suspected secondary EMPD in the anogenital region. The two most common anogenital secondary EMPD-associated internal malignancies are urothelial (CK7+/cytokeratin 20+/GCDFP-15-) and colorectal (CK7+/cytokeratin 20+/GCDFP-) [1,2,12].

Treatment recommendations favor margin-controlled surgery, either MMS or excision with complete circumferential peripheral and deep margin assessment (CCPDMA), as first-line treatment for primary EMPD owing to lower rates of recurrence in comparison to WLE. Alternative therapies when surgery is contraindicated include imiquimod 5% cream, photodynamic therapy, ablative laser, and radiotherapy [1,4]. EMPD’s five-year overall survival rate has been reported between 75% and 85%, with primary EMPD patients generally doing better than those with secondary EMPD [12].

## Conclusions

EMPD on the face is very rare. Along with tumor cells' CK7 positivity, background keratinocytes appropriately staining with P63 help differentiate EMPD from cSCC. This was important in our case, in which the patient had a prior cSCC 1 cm from their EMPD lesion, and the EMPD lesion initially appeared consistent with actinic keratosis. Regardless of GCDFP-15 status, all patients with ectopic primary EMPD should have a malignancy-oriented review of systems performed and be referred for appropriate internal malignancy screening. There is evidence that margin-controlled surgery results in lower recurrence rates than WLE. We report the fifth case of ectopic EMPD arising on the face, and the first EMPD to be reported on the forehead. We hope this case will add to the differential diagnoses physicians consider when examining cutaneous neoplasms on the face.
